# Human Suprapatellar Fat Pad-Derived Mesenchymal Stem Cells Induce Chondrogenesis and Cartilage Repair in a Model of Severe Osteoarthritis

**DOI:** 10.1155/2017/4758930

**Published:** 2017-07-09

**Authors:** Ignacio Muñoz-Criado, Jose Meseguer-Ripolles, Maravillas Mellado-López, Ana Alastrue-Agudo, Richard J Griffeth, Jerónimo Forteza-Vila, Ramón Cugat, Montserrat García, Victoria Moreno-Manzano

**Affiliations:** ^1^Unidad de Traumatología, Hospital Casa de la Salud, Valencia, Spain; ^2^Neuronal and Tissue Regeneration Lab, Centro de Investigación Príncipe Felipe, Valencia, Spain; ^3^Molecular Pathology and Translational Research in Oncology, Unidad Mixta Universidad Católica de Valencia y Centro de Investigación Príncipe Felipe, Valencia, Spain; ^4^Unidad de Artroscopia y Unidad de Traumatología del Hospital Quiron, Barcelona, Spain; ^5^Fundación García Cugat, Barcelona, Spain; ^6^Universidad Católica de Valencia, Valencia, Spain; ^7^FactorStem Ltd., Valencia, Spain

## Abstract

Cartilage degeneration is associated with degenerative bone and joint processes in severe osteoarthritis (OA). Spontaneous cartilage regeneration is extremely limited. Often the treatment consists of a partial or complete joint implant. Adipose-derived stem cell (ASC) transplantation has been shown to restore degenerated cartilage; however, regenerative differences of ASC would depend on the source of adipose tissue. The infra- and suprapatellar fat pads surrounding the knee offer a potential autologous source of ASC for patients after complete joint substitution. When infrapatellar- and suprapatellar-derived stromal vascular fractions (SVF) were compared, a significantly higher CD105 (+) population was found in the suprapatellar fat. In addition, the suprapatellar SVF exhibited increased numbers of colony formation units and a higher population doubling in culture compared to the infrapatellar fraction. Both the suprapatellar- and infrapatellar-derived ASC were differentiated in vitro into mature adipocytes, osteocytes, and chondrocytes. However, the suprapatellar-derived ASC showed higher osteogenic and chondrogenic efficiency. Suprapatellar-derived ASC transplantation in a severe OA mouse model significantly diminished the OA-associated knee inflammation and cartilage degenerative grade, significantly increasing the production of glycosaminoglycan and inducing endogenous chondrogenesis in comparison with the control group. Overall, suprapatellar-derived ASC offer a potential autologous regenerative treatment for patients with multiple degenerative OA.

## 1. Introduction

Osteoarthritis (OA) is a degenerative joint disease with no efficient treatment due to limited endogenous regenerative capacity. It affects the knees of nearly a quarter of the population aged 60 and older [1, 2]. Currently, patients with severe OA are inexorably relegated to prosthetic joint substitution. OA is a disorder involving movable joints characterized by cell stress and extracellular matrix degradation initiated by micro- and macroinjury that activates maladaptive repair responses including proinflammatory pathways of innate immunity. The disease is first manifested as a molecular derangement (abnormal joint tissue metabolism) followed by anatomic and/or physiologic derangements (characterized by cartilage degradation, bone remodelling, osteophyte formation, joint inflammation, and loss of normal joint function) culminating in disease [3, 4].

Since the OA pathological processes are well described involving among others, the loss of functional chondrocytes, several cell-based therapeutic approaches have already been successfully developed including bone-marrow stimulation [5], implantation of osteochondral autograft [6] or allografts (ACI) [7], and transplantation of expanded autologous chondrocytes [8], or amplified mesenchymal stem cells (MSCs) [9], which help to restore articular cartilage. The intra-articular administration of MSC directly in the synovial fluid has been the predominant cell-based approach, with already demonstrated clinical effectiveness, with probed regenerative and immunosuppressant activities [9, 10]. To date, it continues to be an important avenue of research and clinical development due to its extraordinary therapeutic aptitude. However, the direct differentiation of multipotent MSC into cells of the chondrogenic lineage has led to a variety of experimental strategies to investigate whether tissue-specific MSCs are preferential for the regeneration and maintenance of articular cartilage [9]. Additional efforts on engineered cartilage implants are also done by using MSC synergistically activated with biomolecules to potentially improve chondral and osteochondral lesion repair, converting those in specialized trophic producers to initiate endogenous regenerative activities in the OA joint [11–13]. The long-term durability, increased tissue integration, and specific activity depend first on better transplantation survival rates and better adaptation to the hostile environment which still comprises a challenge. To date, there is not a precise description of which donor sample would be more efficient in the generation of MSC for the treatment and repair of joints in a degenerative process. Subcutaneous fat tissue is the most accessible source; however, after prosthetic implementation for joint substitution, the supra- and infrapatellar fat pads are commonly resected, constituting a suitable autologous adipose-derived MSC source (ASC). The infrapatellar or Hoffa's fat pad is an intracapsular but extrasynovial structure, situated in the knee under the patella, between the patellar tendon, femoral condyle, and tibia plateau [14]. The suprapatellar or quadricep fat pad is externally interposed between the joint capsule and the synovium, lined to the joint cavity showing a triangular shape and extended through the patellar base [15]. Previous studies have already described the potential regenerative capability of ASC derived from the infrapatellar pad in a model of OA [16, 17], showing in fact a higher percentage of immunophenotypical positive stromal cells in comparison with those obtained from subcutaneous fat [18]. However, no previous data has been reported regarding the suprapatellar tissue regenerative capacity. In addition, recent reports also focus on the role of the Hoffa's-derived cells, such as inflammatory cells releasing or inducing the release of inflammatory mediators such as IL-6, IL-8, TNF*α*, and PGE2 when derived from OA patients, suggesting that the infrapatellar fat pad is an active joint tissue in the initiation and progression of knee OA [19–22].

There are stem cell niches with no spontaneous capacity to mobilize to the injury to repair the severe OA defects. The amplification of these cells from these specialized sources could render an optimal source of autologous or even allogenic applications to promote cartilage regeneration. Indeed, the patellar fat pad-derived ASC would offer a potential autologous regenerative treatment for patients with multiple degenerative OA.

## 2. Material and Methods

### 2.1. Adipose Tissue Processing, SVF Isolation, and ASC Culture

Twenty-four patients between 50 and 80 years old indicated for complete joint substitution were included in the study. All patients were asked to sign an informed consent for the use of surplus fat tissue in the prosthesis surgery as well as a donation of peripheral blood (~20 ml) for autologous serum isolation. The samples, adipose tissue from supra- or infrapatellar areas, were anonymized and individually housed and collected inside the surgery room in sterile containers with sterile saline. This experimental procedure has been evaluated and accepted by the Regional Ethics Committee for Clinical Research with Medicines and Health Products following the Code of Practice 2014/01. As exclusion criteria, no samples were collected from patients with a history of cancer and infectious diseases active at the time of the surgery (viral or bacterial).

The adipose tissue, from supra- or infrapatellar areas, was transferred from the surgery room to the laboratory in a hermetic container at 4°C in sterile solution. The samples were washed multiple times in PBS plus antibiotics to clean the tissue and remove residual blood. The samples were distributed within 10 g of adipose tissue per 100 mm petri dish, with a solution containing PBS, 100 units/ml penicillin and 100 *μ*g/ml streptomycin (Gibco 15,140), collagenase type IA (0.07%, Sigma C9891 CA, USA), and dispase I (0.2 mM Sigma). The tissue was cut into small pieces using sterile surgical scissors in a laminar flow hood and digested in a closed cell flask overnight in a shaker at 37°C, 20% O_2_, 5% CO_2_. The following day, the digested adipose tissue was collected and washed multiple times with PBS plus antibiotic by serial centrifugation. The cell pellet constitutes the stromal vascular fraction (SVF) and was suspended in 1 ml of PBS for cell counting via a Neubauer® chamber. For FACS analysis, 10^5^ cells were utilized for each pair of antibodies. The remaining cells were employed for cell amplification and posterior analysis, distributed in two groups, growth in 10% human serum containing medium or 10% fetal bovine serum (in DMEM medium containing 2 mM L-glutamine, 30% L-glucose, 100 units/ml penicillin, and 100 *μ*g/ml streptomycin), plated in petri dishes, and incubated overnight. The following day, the medium was removed and replaced with fresh medium and attached cells were allowed to grow until nearly confluent and then subjected to cell proliferative analysis, clonicity assay, morphological assessments, and FACS analysis and cell differentiation assays.

### 2.2. FACS Analysis

The SVF was assayed for cell surface protein expression by flow cytometry (FC500, Beckman Cultek, USA). 10^5^ cells, diluted into 100 *μ*l of PBS were incubated with 1 : 50 dilution for every pair of antibodies: CD90-PE and CD29-APC, CD44-PE-Cy, CD117-APC, CD105-PE, CD45-FITC, and CD34-PE (BD Pharmigen, USA). As a negative control, cell suspension without antibody was employed following the same procedure. Cells were incubated in the dark for 45 min at room temperature and then washed three times with PBS and suspended in 0.3 ml of cold PBS for FACS analysis. The mean ± SD of every identified population (in percentage) of all tested samples was determined.

### 2.3. Colony Formation Assay

The amplification and expansion of the ASC population from the SVF involves first the colony forming units after attachment onto a substrate and then a subsequent amplification in the presence of appropriate growth factors [23]. Fifty cells at passage three of every sample, supra- and infrapatellar, of four different patients were seeded in 6-well plates in the presence of 10% human serum or 10% fetal bovine serum containing medium for 10 days. The medium was replaced every third day. Subsequently, the cells were fixed with cold methanol for 5 minutes and washed with PBS before incubating in Giemsa solution (Sigma, USA) for 30 minutes. The excess stain was removed by subsequent washes with tap water. The cells were allowed to air dry and then visualized under the microscope. The mean ± SD of the total number of colonies at each condition of all tested samples was determined.

### 2.4. ASC Proliferative Analysis

At passage three of every sample, supra- and infrapatellar, 10^4^ cells were seeded in 24-well plates in the presence of 10% human serum or 10% fetal bovine serum containing medium. Cells were seeded for up to ten days of analysis, by quantifying the number of cells in a Neubauer chamber every day in every well. The mean ± SD at every time point (every consecutive day) was represented.

### 2.5. Transmission Electron Microscopy

Cells were seeded at 2000 cells/cm^2^ in Lab-Tek chamber slides of 2 wells (Nalge Nunc International, Naperville, IL) and were fixed in 3% glutaraldehyde for 1 hour at 37°C. Cells were postfixed in 2% OsO4 for 1 hour at room temperature and stained in 1% uranyl acetate in the dark for 2 h at 4°C. Finally, cells were rinsed in distilled water, dehydrated in ethanol, and infiltrated overnight in Durcupan resin (Fluka, Sigma-Aldrich, St. Louis, USA). Following polymerization, embedded cultures were detached from the chamber slide and glued to araldite blocks. Serial semithin sections (1.5 *μ*m) were cut with an Ultracut UC-6 (Leica, Heidelberg, Germany) and mounted onto slides and stained with 1% toluidine blue. Selected semithin sections were glued with Super Glue-3, Loctite (Henkel, Düsseldorf, Germany), to araldite blocks and detached from the glass slide by repeated freezing (in liquid nitrogen) and thawing. Ultrathin sections (0.06–0.08 *μ*m) were prepared with the Ultracut and stained with lead citrate. Finally, photomicrographs were obtained under a transmission electron microscope FEI Tecnai G2 Spirit (FEI Europe, Eindhoven, Netherlands) using a digital camera Morada (Olympus Soft Image Solutions GmbH, Münster, Germany).

### 2.6. ASC Directed-Differentiation

ASC at or after passage 4 were subjected to directed differentiation [24], to induce adipogenesis, osteogenesis, and chondrogenesis, for each sample, supra- and infrapatellar from three different patients. All directed-differentiation media were obtained from Lonza (Lonza Co., Basel, Switzerland). *Adipogenesis*: ASC were seeded at a cell density of 10,000 cells/cm^2^ and when ASC were >90% confluent, the growth medium was substituted for differentiation medium containing insulin, dexamethasone, IBMX (3-isobutyl-methyl-xantine), and indomethacin (adipose-derived stem cell basal medium; Lonza Co.). The cells were then incubated in standard cell culture conditions for 12 days. The adipogenic differentiation was evaluated by Oil Red staining of the lipid vacuoles in formalin-fixed cultures; Oil Red O stock solution (5 g/l of isopropanol) was diluted 6/10 in water and incubated for 1 hour at RT. Serial washes with water to remove the excess staining were applied. After bright-field picture acquisition, total Oil Red O stain was extracted with 100% isopropanol for 5 min with gentle rocking and the absorbance at 492 nm was quantified. The mean ± SD of the absolute numbers at each condition of all tested samples was determined. *Osteogenesis*: ASC were seeded at a cell density of 10,000 cells/cm^2^ in collagen I (Sigma; 10 mM) coated plates in medium containing 0.1 *μ*M dexamethasone, 50 *μ*M Asc2P, and 10 mM *μ*-glycerophosphate (osteogenic basal medium; Lonza Co.) with 10% human serum. ASC cultures were maintained in this medium for 4 weeks (with medium changes every 3 days). For detection of extracellular calcium deposits, Alizarin red staining was used in formalin-fixed cultures; Alizarin red solution (0.2 g/l water) was incubated for 2-3 minutes, until the reaction was observed microscopically. Excess dye was removed by several washing steps using water. The orange-red calcium precipitates were quantified from at least four different pictures of each sample by using ImageJ software. *Chondrogenesis*: The ASC culture was performed from cell “Micromass” starting with a high concentration of cells in a minimal volume (1 × 10^5^ cells/100 *μ*l) in the presence of TGF-*β* 1 and 3 (10 ng/ml), Asc 2P (50 *μ*M), and insulin (6.25 *μ*g/ml) (Chondro BulletKit; Lonza Co.) for four weeks with medium changes every 3 days. Alcian blue (0.1 g/l in water, pH 1.0) was used to detect the presence of enrichment of sulphated proteoglycans in the extracellular matrix. Before staining, the micromass cultures were fixed in formalin, embedded in paraffin, and sectioned into 10 *μ*m. Blue staining was quantified from 4 different pictures of each sample with ImageJ software.

### 2.7. OA Mouse Model and Cell Transplantation

Twelve-week old adult male C57BL6 mice were experimentally induced to have severe OA in both knees by a single injection of 13 U collagenase II in 6 *μ*l of 5 mM CaCl_2_ [25]. Five days later, when the OA by severe cartilage damage was induced, all animals were distributed into 4 groups (*n* = 6 per group). The *control* group was injected with 6 *μ*l of growth cell culture medium; 10^5^ cells in 6 *μ*l of SVF or hASC (from suprapatellar fat pad) were injected in the SVF or hASC groups, respectively. Six *μ*l of thawed pooled human platelet rich plasma (PRP) was injected in the PRP group. All intra-articular injections were performed by using a 30G Hamilton syringe connected to a Remote Infuse/withdraw pump 11 elite nanomite programmable syringe pump (Harvard Apparatus) at a 2 *μ*l/min infusion rate. The PRP was isolated from blood collected from all patients in citrate tubes and was prepared following the standardized method described in Anitua et al. [26] and pooled to minimize individual modifications. All animals were sacrificed one month after OA induction, and knee joints were collected. The experimental protocol was approved by the Animal Care Committee of the Centro de Investigación Principe Felipe (Valencia, Spain) in accordance with the National Guide to the Care and Use of Experimental Animals (Real Decreto 53/2013). The leg was isolated and osteomized in the middle of the femoral shaft and in the middle of the tibia and fibula to get the whole joint. Most of the adherent connective tissue, including muscle, ligaments, and tendons, surrounding the knee were removed preventing any damage to the cartilage. One knee was used for histological analysis and the other knee for the quantification of glycosaminoglycans (GAGs).

### 2.8. GAG Analysis

Immediately after tissue dissections, GAGs were individually quantified for tibia and femur portions from one treated knee of all animals in all groups. Weighted samples were digested in 2.5% papain solution by overnight incubation at 65°C. The supernatant was incubated with 10% Alcian blue working solution containing 0.25% of Triton X-100 in 18 mM H_2_SO_4_ and then incubated with dissociation solution (4 M guanidine HCl, 0.375 Triton X-100 in 0.027 M H_2_SO_4_) for 30 min. The precipitated GAGs were suspended in 8 M guanidine HCl and quantified at 570 nm. A chondroitin sulfate standard curve was prepared in parallel.

### 2.9. Safranin-O Staining and Histological OA Scoring

Bones fixed in 4% PFA were incubated for 7 days in 0.1 M ethylene diamine tetra-acetic acid (EDTA) for decalcification before paraffin embedding and frontally sectioning the entire knee into 5 *μ*m sections. Deparaffinized and hydrated slices were first stained for 5 min with hematoxylin (Sigma) and, after several washes, quickly destained in acid EtOH, stained with fast green (0.001%) solution for 5 minutes, quickly rinsed with 1% acetic acid solution, and then stained with 0.1% safranin-O solution for 5 minutes. Dehydrated samples were mounted in Eukit. Bright-field pictures of all series (every 4th section) were acquired for OA score quantification following the recommendations established by Glasson et al. [27]. The entire joint was analysed at all four quadrants and through multiple step sections through the joint following the 0–6 subjective scoring system, where 0 is normal; 0.5 means loss of safranin-O without structural changes; 1, small fibrillations without loss of cartilage; 2, vertical clefts down to the layer immediately below the superficial layer and some loss of surface lamina; 3, vertical clefts/erosion to the calcified cartilage extending to <25% of the articular surface; 4, vertical clefts/erosion to the calcified cartilage extending to 25–50% of the articular surface; 5, vertical clefts/erosion to the calcified cartilage extending to 50–75% of the articular surface; and 6, vertical clefts/erosion to the calcified cartilage extending to >75% of the articular surface.

### 2.10. Immunoassay

Both fixed cells on a cover slip from osteogenic-directed differentiation or the chondrogenic-directed differentiated as well as fixed bone (after demineralization by EDTA immersion) paraffin-embedding sections were subjected to protein expression analysis by specific immunostaining. The paraffin-embedded sections were first deparaffinized. Both were fixed with 4% paraformaldehyde at room temperature for 10 min and washed with PBS, permeabilized with a PBS solution containing 0.1% Triton X-100, and blocked with 5% goat serum in PBS for 1 h. The following primary antibodies were diluted in blocking solution and incubated overnight at 4°C. Polyclonal rabbit *α*-Sox9 (Millipore (1 : 200) or monoclonal mouse *α*-Connexin 43 (Invitrogen; (1 : 200). After being rinsed three times with PBS, the cells were incubated with Alexa647 dye conjugated goat anti-mouse IgG (1 : 400; Invitrogen, CA, USA) secondary antibody and Phalloidin-Alexa 488 (Invitrogen, CA, USA) for 1 h at room temperature. Tissues were incubated with Oregon Green-488 dye conjugated goat anti-rabbit IgG (1 : 400; Invitrogen, CA, USA) secondary antibody. All samples were counterstained by incubation with 4,6-diamidino-2-phenylindole hydrochloride (DAPI) from Molecular Probes (Invitrogen, USA) for 3 min at room temperature followed by washing steps. Samples were mounted using Fluor Save Reagent (Calbiochem, USA). Signals were visualized by confocal microscopy (Leica, Germany); at least 6 different fields per condition and assay were analysed.

### 2.11. PET Analysis

For PET image acquisition (Albira Si PET subsystem; Oncovision, Spain), an additional group of animals was included. After OA induction, the right knee was injected with medium (control) and the left knee was injected with 10^5^ hASC in 6 *μ*l each, in order to minimize individual differences of the NaF-18 probe signal distribution and acquisition. The probe was i.v. injected though the tail with a range of activities between 177 and 199 uCi with uptake times that varied between 83 and 110 min. In all cases, the whole body PET scan time was 10 minutes. The images were analysed by using Amide® software. The mean ± SD of the ROIs at each condition of all animals was determined.

### 2.12. Statistical Analysis

Statistical comparisons were assessed by Student's *t*-test. All *P* values were derived from a two-tailed statistical test using the SPSS 11.5 software. A *P* value <0.05 was considered statistically significant.

## 3. Results

### 3.1. Suprapatellar Fat Pad SVF Is Enriched in CD105 Positive Cells

**Figure 1:**
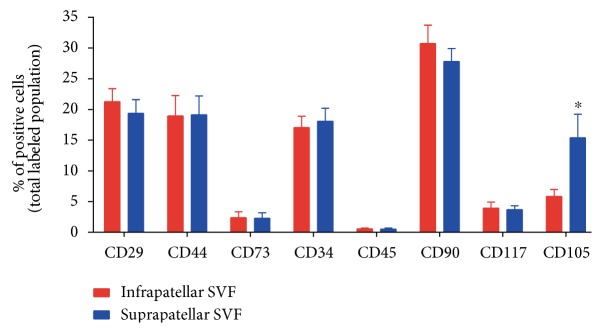
Supra- and infrapatellar fat pad cell populations in the SVF. The SVF derived from the mechanical and enzymatic homogenization of each fat pad of the knee were subjected to FACS analysis for immunodetection of mesenchymal superficial cell markers CD29, CD90, CD44, CD117, and CD105 as well as the hematopoietic cell marker CD34 and the mature leukocyte marker CD45. The positive cell populations were expressed as a percentage of the total analysed cell population. The mean ± SD of the identified population (in percentage) of twenty-four different samples was shown. ^∗^*P* < 0.05 in comparison with infrapatellar group.

The supra- and infrapatellar fat pads of twenty-four patients with severe OA were resected during the surgical intervention for prosthetic implantation. After tissue processing, total cell counting rendered no significant differences in the amount of nucleated cells between the supra- or infrapatellar samples (7.8 × 10^5^ ± 2.8 × 10^5^ infrapatellar cells versus 8.8 × 10^5^ ± 4.4 × 10^5^ suprapatellar cells/g of adipose tissue; Supplementary Figure available online at https://doi.org/10.1155/2017/4758930). The immunophenotypic analysis of the SVF showed in both cases a positive population for a panel of typical human mesenchymal cell markers including CD90, CD29, CD44, CD117, and CD105 (Figure 1) [28]. Interestingly, the suprapatellar-derived SVF contained a significant enrichment of CD105(+) cells in comparison with the infrapatellar-derived cell suspension (Figure 1). No significant differences were detected in any other assayed marker, including the hematopoietic precursor cell marker CD34 or the mature leucocyte cell marker CD45.

### 3.2. The Suprapatellar ASC Show a Faster Proliferative Profile

**Figure 2:**
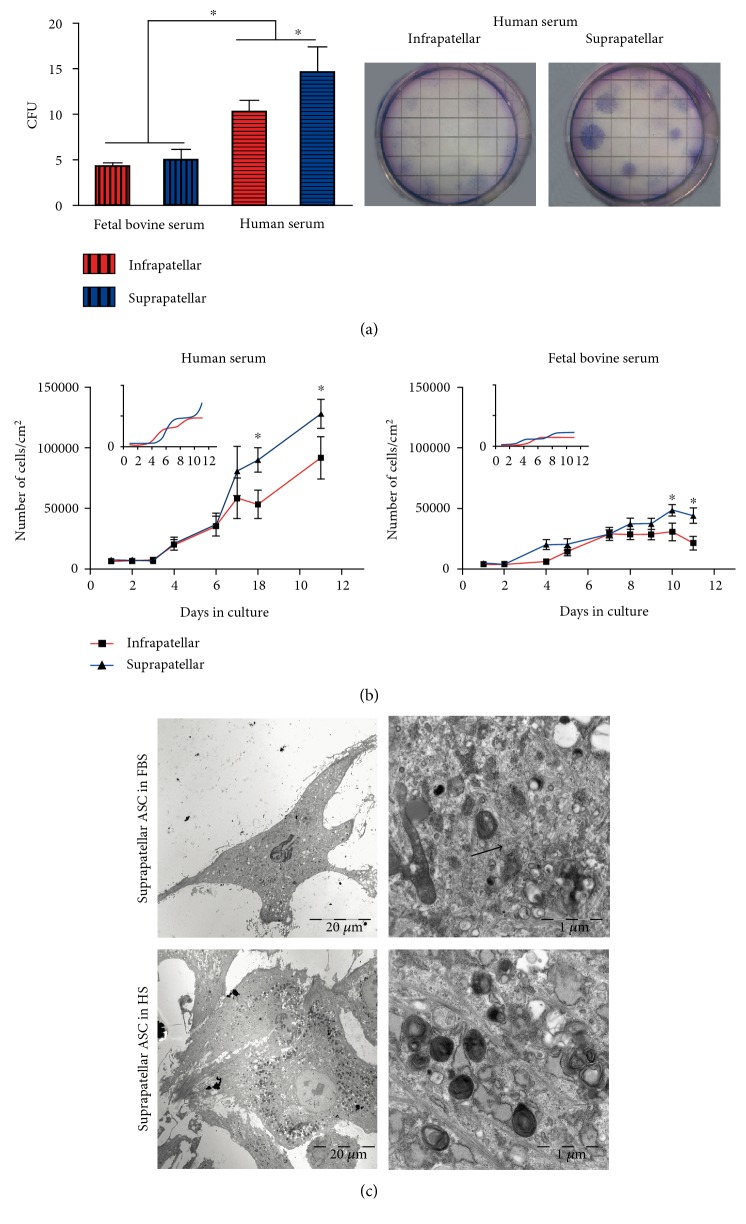
Supra- and infrapatellar ASC proliferative activity. (a) Colony-forming unit (CFU) assay was performed from infra and suprapatellar SVF from four different patients in the presence of 10% human serum (HS) or 10% fetal bovine serum- (FBS-) containing medium for 10 days. The generated colonies were counted after Giemsa staining (right panel). The mean ± SD of the total number of colonies at each condition of all tested samples was shown. ^∗^*P* < 0.05 at the indicated comparisons. (b) 10^4^ cells at passage three of every sample, suprapatellar (blue line)- and infrapatellar (red line)-derived ASC were allowed to grow in multiple wells in the presence of 10% HS or 10% FBS and quantified every day up to 10 days. The mean ± SD of the total number of cells at every time point was represented. ^∗^*P* < 0.05 versus infrapatellar. (c) Representative images after TEM acquisition of suprapatellar ASC growth in HS (left panels) or FBS (right panels) are shown. White arrows: inclusion bodies; black arrows: intermediate filaments.

ASC cultures derived from both supra- and infrapatellar tissues were allowed to reach cell confluency and were kept for three passages before cell proliferative analysis in the presence of human serum (HS) or fetal bovine serum (FBS). For colony-forming unit (CFU) quantification, a clonogenic assay was performed (Figure 2(a)). When ASC were grown in the presence of FBS, no differences were found. However, when ASC were grown in the presence of HS, there was a significant increase in the number of CFU and indeed the suprapatellar-derived ASC showed a higher CFU in comparison with the infrapatellar fraction (Figure 2(a)). The colonies formed from the suprapatellar SVF were not only more abundant, but they were always bigger (Figure 2(a), right panel) and exhibited a faster proliferative profile. In fact, when the ASC were cultured with either FBS or HS, the proliferative rates, in terms of the number of cells per growth area, within a cell growth curve daily analysis, were significantly higher from those cells amplified from the suprapatellar fat tissue compared with infrapatellar. The differential growth rate was significantly different eight days after culture in the presence of HS and ten days after culture in FBS (Figure 2(b)). The ultrastructural analysis of the suprapatellar cell cultures grown in FBS or HS showed higher inclusion body accumulation (indicated by white arrows) and higher distribution of intermediate filaments (indicated by black arrows) in the presence of HS-containing medium (Figure 2(c)).

### 3.3. In Vitro Osteogenesis and Chondrogenesis Are Favored in Suprapatellar ASC

**Figure 3:**
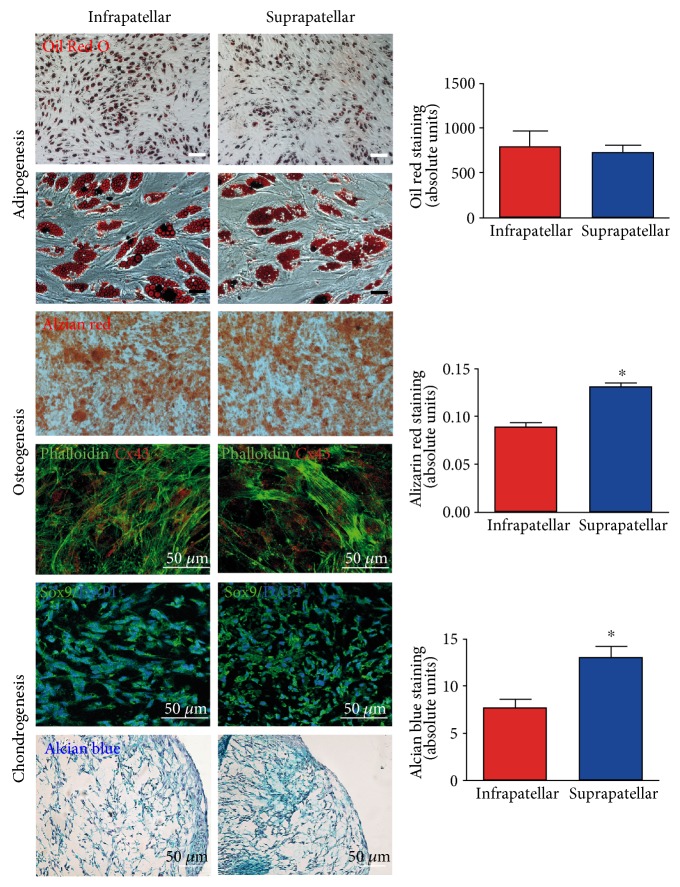
Multilineage potential of suprapatellar- and infrapatellar-derived ASC. All directed differentiation processes were induced from both samples, suprapatellar- and infrapatellar-derived ASC at passages after 4. To quantify the efficiency of cell differentiation, specific staining was employed for each process: Oil Red O staining for adipogenesis, Alizarin red for osteogenesis and Alcian blue for chondrogenesis, ^∗^*P* < 0.05 versus infrapatellar. In addition, immunodetection of Connexin 43 (red; osteocyte marker) and phalloidin (green; cytoskeletal marker) was performed to evaluate osteocyte maturation. Sox9 (green) counterstained with DAPI (blue, nucleus) stained newly generated chondrocytes.

To further assess the stem cell characteristics from both adipose tissue sources, the supra- and infrapatellar fat pads, we compared the differentiation multipotency between both samples (Figure 3). Both underwent induced differentiation into the adipogenic, osteogenic, and chondrogenic lineages (Figure 3). However, the extent of differentiation was not identical. Suprapatellar-derived ASC showed higher osteogenic and chondrogenic potential in comparison with the infrapatellar-derived and infrapatellar-differentiated cells as indicated by quantification of Alizarin red and Alcian blue staining, respectively (Figure 3, right panels). The higher chondrogenic potential of the suprapatellar-derived cells was also supported by the increased expression of Sox9 (green), a chondrocyte precursor marker [29]. This differential multipotency yields the suprapatellar fat pad a promising cell source for cartilage tissue repair.

### 3.4. Suprapatellar-Derived ASC Significantly Improves Cartilage Regeneration in a Mouse Model of Severe OA

To assess whether the increased cell proliferative and cell differentiation properties shown in vitro of the suprapatellar-derived MSCs would be functionally efficient in cartilage regeneration, in vivo cell transplantation assay in a severe OA mouse model was performed.

**Figure 4:**
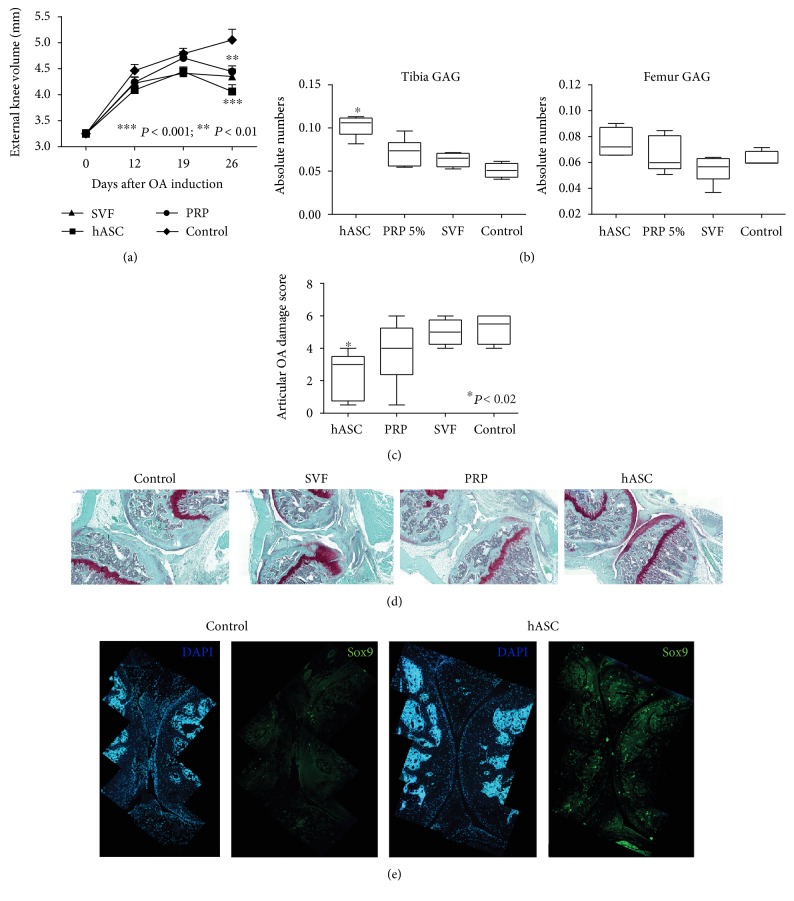
Functional and structural cartilage regeneration. The therapeutic activity of suprapatellar-derived ASC (supra-hASC) in comparison with control-, SVF-, and PRP- treated groups was studied in a mouse model of severe OA. (a) Swelling-related inflammation was measured by quantifying once a week the external volume of the knees, ^∗∗^*P* < 0.01^∗∗∗^*P* < 0.001 versus control. (b) Glycosaminoglycan (GAG) quantification at both, tibia and femur, ^∗^*P* < 0.05 versus control. (c) Articular OA damage score was performed from 5 *μ*m frontally sectioned knees stained with safranin-O and following the recommendations established by Glasson et al. [27]. ^∗^*P* < 0.05 versus control. Representative images of a frontal section stained with safranin-O are shown for each group. (d) Immunostaining of Sox9 (green) to evaluate the endogenous chondrogenic activity. All cell nuclei were detected with DAPI staining (blue).

For the therapeutic efficacy testing of the suprapatellar-derived human ASC (hASC), severe OA was induced by single intra-articular injection of collagenase II (13 U) in both knees of 12-week old adult male C57BL6 mice. Five days after injection, when total cartilage destruction occurs (previously evaluated by histological analysis; data not shown), 10^5^ hASC or the SVF or growth medium (control) was injected into the intra-articular space. It has been shown that PRP infiltration, increasingly implemented in regular clinical practice, can reduce pain and improve joint function with a notable improvement on the quality of life of patients [30, 31]. Therefore, PRP was also injected in an additional group of animals (PRP) in order to compare the regenerative effectiveness on cell transplantation in comparison with growth factor infiltration (Figure 4).

Once a week after treatments, the volume of the knees was measured using an automatic caliper. As shown in Figure 4(a), a significant increase in knee size occurs in all three groups after OA induction during the whole experiment. However, all treated groups, hASC, SVF, and PRP, showed a significant reduction of this increased volume one month after OA induction compared to the control group (Figure 4(a)). This result indicates a significant anti-inflammatory effect of the treatments. However, when we examined the structural regenerative effect by quantifying GAG (Figure 4(b)) and the OA damage score (Figure 4(c)), we observed a unique consistent structural regeneration of the loss of cartilage when the animals were treated with hASC in comparison with the control group (Figures 4(b) and 4(c)). Thus, PRP or SVF acts as an effective anti-inflammatory agent, but the regenerative effects of the stem cells do not seem to be bolstered by PRP or SVF. A representative safranin-O staining image for each treated group showed a complete absence of articular cartilage in the control animal and safranin-O-positive areas compatible with regenerating cartilage more prominent in the hASC-treated animal (Figure 4(d)).

To explore the contribution of the ectopic ASC to the regeneration of the articular cartilage, we evaluated the induced chondrogenic activity by the detection of Sox9 (a chondrocyte progenitor marker) positive cells in the control and supra-hASC-treated joints (Figure 4(e)). As shown in the representative images, an increase in Sox9 positive cells was detected in the hASC group (Figure 4(e)). However, few of the Sox9 positive cells costained with the specific immunodetection for human cells (antinuclei; data not shown) indicating an endogenous induction of the cartilage repair by the hASC transplantation.

### 3.5. Positron Emission Tomography (PET) Analysis Shows In Vivo Cartilage Regeneration Process after ASC Trasplantation

**Figure 5:**
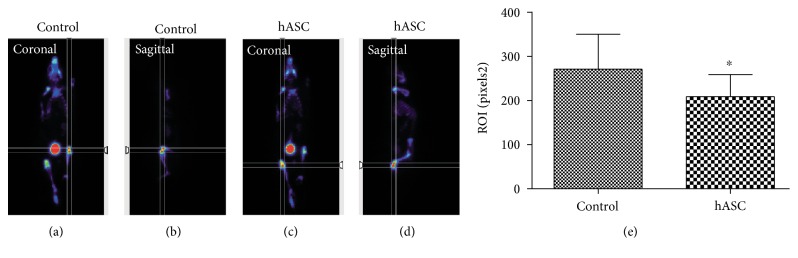
Translational PET image analysis after hASC transplantation in the OA mouse model. PET acquisition (a, c) and quantification (e) of NaF-18 detection of the control knee (a, b) and hASC transplanted knee (c, d). The images were analysed by using Amide software. The mean ± SD of the region of interest at each condition of all animals was determined. ^∗^*P* < 0.05 versus control.

18F-NaF PET/CT is a high-performance diagnostic tool utilized by clinical practitioners and produces high-quality images within a shorter period from injection time in bone and joint disorders including osteoarthritis [32, 33]. PET analysis was employed to evaluate cartilage regeneration in vivo in a group of animals with induced OA in both knees. In order to minimize the individual differences of the NaF-18 probe signal distribution and acquisition (injected one month after OA induction), both conditions were included in the same animal; the left knee was transplanted with hASC and the right knee served as a control (Figure 5). After quantification of the ROIs from the acquired images at equivalent coronal and sagittal planes, a significantly lower detection of NaF-18 on the joints transplanted with hASC (Figure 5, right graph) compared to the control joints was observed. The bone at the hASC transplanted joints was less exposed, and the signal was masked by the regenerated cartilage.

## 4. Discussion

ASC-based therapies have already been tested in clinical trials, at the safety and efficacy phases, with remarkable restorative activity in the treatment of OA from subcutaneous abdominal fat [34, 35]. However, tissue-specific-derived stem cells would guarantee the improvement of ASC repair capabilities for instance, in terms of improved chondrogenic efficiency for cartilage repair [9]. In the knee, there are two major sources of ASC, the supra- and infrapatellar fat pads [14, 15]. Both fat pads are commonly resected during knee joint arthroplasty in patients with acute OA and are a suitable source of ASC for the autologous treatment of additional affected joints. Previous studies have already described the potential regenerative capability of ASC derived from the infrapatellar pad, recently shown to have demonstrated increased chondrogenic potential compared with those from subcutaneous fat in vitro [36] and in vivo in a model of OA [16, 17, 37]. However, no data have been reported yet with the suprapatellar-derived cells. Here, we show for the first time the regenerative capacity of the suprapatellar-derived ASC population in a severe OA mice model. The infrapatellar pad-derived cells have been considered an active joint tissue in the initiation and progression of knee OA, activating the inflammatory cells by producing inflammatory mediators influencing the cartilage and synovium metabolism [38]. When we compare in vitro the proliferative and differentiated properties among both derived ASCs, we demonstrate improved proliferative, chondrogenic, and osteogenic rates from the suprapatellar-derived ASC, showing a promising and improved source for efficient cell therapeutic resolution of joint degeneration [33].

The CD105 or endoglin receptor has been related to the more proliferative, migrating, and invading MSC size populations in the injury area during endogenous regeneration [39]. Moreover, transplantation of a CD105-negative stem cell population showed significant chondroid degeneration features compared to the CD105-positive transplanted group [40], showing an influence on chondrogenic cell fate induction. In fact, when we compare the directed chondrogenic capability between the two samples, we found a better yield from the suprapatellar-derived ASC that shows an enrichment of CD105-positive population versus those from the infrapatellar fat.

The ASC amplification in vitro with human serum was always better than with fetal bovine serum. Previous reports from subcutaneous adipose tissue have already shown detrimental proliferative activity of ASC in the presence of FBS [41]. Differential gene expression analysis of ASC cultured with HS or FBS showed a significant overexpression of a number of genes related with regulatory roles in cell cycle progression [42].

For translational and functional proof of concept, an in vivo model of severe OA [25] for intra-articular administration of the hASC was explored comparing the efficiency of cartilage regeneration in the SVF- and PRP-treated animals. Interestingly, all treated groups showed significantly reduced knee joint diameter (swelling).

PRP contains a multitude of growth factors including TGF-*β*, PDGF, IGF, bFGF, VEGF, and EGF [43] with anabolic chondral-promoting and chondral-protective properties [44, 45]. Abundant preclinical and clinical findings support that PRP is a promising coadjutant treatment for cartilage repair and relieving symptoms based on its anabolic effect on the resident cells and due to its potential to inhibit inflammation and alleviate OA symptoms with a clinically acceptable safety profile [46]. PRP treatment has shown additional benefits in the regeneration of other related tissues such as bone defects, indicating PRP plays a crucial role by influencing the local tissue microenvironment and thereby enhancing progenitor cell recruitment and proper matrix deposition [47]. Although only the ASC transplantation showed significant tissue regeneration, the PRP combination could create a synergistic effect by bridging and interfacing between the MSCs and the endogenous tissue, an effect not produced by an individual treatment alone. Chemically induced OA in the mice led to aggressive joint degeneration; however, even in such a severe condition, ASC transplantation reduced and rescued functional anatomy in the intra-articular space, reducing the OA damage scores and increasing the cartilage-like tissue. Continued expression of Sox9 is required to maintain hyaline-like cartilage, avoiding chondrocyte hypertrophy for efficient regeneration [29]. Only the amplified hASC were able to induce efficient regeneration one month after treatment showing a significant increase in SOX9 expression in the growing cartilage. One month after cell transplantation, very few human cells were found in the transplanted joints (data not shown), which is in accordance with previous reports [48, 49]. Rather than assuming cell replacement by ASC transplantation, the most contemporary hypothesis is that their actions are mostly associated with paracrine activity by secreting modulating factors. Accordingly, ASC secrete PGE2 which induces dendritic cells to upregulate the anti-inflammatory cytokine IL10 while reducing the proinflammatory TNF*α* and IL12 contributing to its known immune-modulatory effect and related promotion of tissue repair [50, 51]. However, this hypothesis needs to be addressed with further research to demonstrate its relevance for the treatment of OA.

## 5. Conclusion

Our results show that the human suprapatellar fat pad offers a proper ASC source for cartilage regeneration by promoting efficient endogenous chondrogenesis in a mouse model of severe OA.

## Supplementary Material

Twenty-four samples of each fat pad, suprapatellar and infrapatellar, were digested overnight and after erythrocyte lysis, and total cell counting of the resulting stromal vascular fraction (SVF) was performed and normalized to the corresponding tissue weight. The mean ± SD of the total cell number per gram of fat tissue at each condition of all tested samples was determined and represented.
